# Symptomatic and Functional Recovery From Major Depressive Disorder in the Ibadan Study of Ageing

**DOI:** 10.1016/j.jagp.2017.12.011

**Published:** 2018-06

**Authors:** Akin Ojagbemi, Taiwo Abiona, Zhehui Luo, Oye Gureje

**Affiliations:** a World Health Organization Collaborating Centre for Research and Training in Mental Health, Neuroscience, and Substance Abuse, Department of Psychiatry College of Medicine, University of Ibadan, Ibadan, Nigeria; b Department of Epidemiology and Biostatistics, College of Human Medicine, Michigan State University, East Lansing, MI

**Keywords:** Depression, disablement process, recovery trajectory, low- and middle-income countries

## Abstract

**Objectives:**

Very little is known about the association between symptomatic and functional recovery from late-life major depressive disorder (MDD) in sub-Saharan Africa. We investigated factors associated with sustained symptomatic remission (SR) from MDD and the 5-year trajectory of post-MDD physical functioning.

**Design:**

5-year prospective study with three follow-up waves in 2007, 2008, and 2009.

**Setting/Participants:**

Household multistage probability sample of 2,149 Nigerians who were aged 65 years or older.

**Measurements:**

Activities of Daily Living (ADL) and MDD were assessed using the Kadz index and Composite International Diagnostic Interview, respectively. We studied those with current MDD (prevalent in 2003–2004 or incident in 2007), and who achieved SR in subsequent waves compared with a chronic/recurrent course (CR).

**Results:**

Baseline demographic characteristics, health, and lifestyle factors were not associated with SR in logistic regression analyses. In mixed-effect linear regression models adjusting for age, sex, and socioeconomic status, ADL worsened in SR (β = 1.0, 95% CI: 0.2, 1.8), but more so in CR (β = 2.3, 95% CI: 1.6, 3.0). Poorer ADL at follow-up was predicted by age (β = 2.9, 95% CI: 1.8, 4.0) and economic status (β = 1.4, 95% CI: 0.3, 2.4).

**Conclusions:**

There was a deteriorating course of disability despite symptomatic recovery from late-life MDD in this sample. This finding has implications for policy and guidelines for the management of late-life depression and disability.

## Highlights

• Full functional status does not always follow symptomatic recovery from major depressive disorder in community elder Africans.
• In this population, symptomatic recovery from major depressive disorder was associated with younger age, higher education, and healthcare utilization in the past year.
• Poorer physical functioning after symptomatic recovery from major depressive disorder was predicted by age over 80 years and lower economic status.
• Programmes targeting functional disability in the elderly need to be considered, not only in terms of physical disorders, but also mental health disorders like depression.
• Future guidelines for treatment of late-life major depressive disorder should consider rehabilitation for any resulting functional disability

Evidence from clinical trials suggests that, following treatment for late-life depression, functional disability may subsist even after symptomatic recovery.^1^, ^2^ Nevertheless, few prospective studies of large community samples^3^, ^4^ have examined whether, as it is in experimental conditions, recovery of physical functioning in the context of late-life depression follows a different course to symptomatic recovery. Better understanding in this area may be important for the development of interventions that target not only symptoms of late-life depression, but also the associated functional disability.

The knowledge gap on the predictors and course of symptomatic and functional recovery from late-life depression, especially from major depressive disorder (MDD), in persons living in low- and middle-income countries (LMICs) is even more striking. This is because of reports from several of such countries^5^, ^6^ suggesting some of the highest rates of late-life depression and associated disability in the world. Also, as interaction between illness and contextual factors may be important in determining both symptomatic and functional trajectories of diseases,^7^ the profile of MDD can be expected to differ between LMICs and more developed country contexts.

In a previous cross-sectional survey by our group,^8^ late-life MDD in community-dwelling elderly Nigerians was associated with greater impairment in work and home functioning when compared with several chronic medical and pain conditions. In the present study, which is a 5-year prospective follow-up of the same sample,^8^ we aimed to 1) determine the predictors of symptomatic and functional recovery from MDD in community-dwelling elderly Nigerians who were participants in the Ibadan Study of Ageing (ISA) and 2) assess whether recovery of physical functioning in late-life MDD follows a different course to symptomatic recovery. Our main hypothesis was that the functional trajectory of MDD will be different in persons with symptomatic recovery compared with those with a more chronic course of illness, with recovery of physical functioning lagging behind symptomatic recovery.

## Methods

The ISA is a community-based prospective survey of the health and well-being of elderly persons living in communities spread across the Yoruba-speaking southwestern and north-central region of Nigeria, a geographical area inhabited by a population of 25 million at the time of study. The Yorubas are a distinct ethnic group with regard to language, culture, and social organization in Nigeria. Within the context of the country, these regions were relatively better resourced for mental health service, with about three psychiatrists to a population of one million at the time of the study, even though most of these specialists were located in about 6 urban centers.

The methodology of the ISA has been fully described.^9^ Here we provide a brief description of methods of ISA relevant to the objectives of the present study.

### Sample Selection, Recruitment, and Follow-Up

We selected one respondent per household using a multistage stratified area probability sampling of households. When more than one elderly person was eligible for the study (eligibility criteria were being aged 65 years or older and fluent in the language of the study, Yoruba), the Kish table selection method was used to select one respondent.^10^ Respondents were informed about the study, invited to participate, but also assured of their right to decline. Participants were those who provided consent, mostly verbal, either because of illiteracy or by choice, before interviews were conducted. Up to five calls were made to contact the selected individual; and there was no replacement for those who could not be contacted or who refused to participate in the study. After the selection procedures, we conducted baseline assessments, between November 3, 2003, and August 27, 2004, on a total of 2,152 respondents (of which 2,149 were complete). Nonresponse was predominantly due to non-availability after repeated visits (14%), interviewers unable to trace the original address (4%), death (3%), physical incapacitation (2%), and, rarely, due to refusal (2%). Participants were followed up in three annual follow-up waves in 2007, 2008, and 2009.

The survey was approved by the University of Ibadan/University College Hospital Ibadan joint ethics review board.

### Measures

Face-to-face interviews were carried out in the homes of participants to assess a range of domains including sociodemographic details, economic positions, social contacts with family/friends, engagement in family/community activities, MDD, dementia, chronic medical and pain conditions, physical disability, and lifestyle risk factors.

### Ascertainment of Major Depressive Disorder

Assessments for MDD were carried out in 2003–2004 and at each follow-up wave using the World Mental Health Survey version of the WHO Composite International Diagnostic Interview (CIDI)^11^ and diagnosed according to the DSM-IV criteria.^12^ The DSM-IV organic exclusion rules were imposed in making the diagnosis of MDD as required by convention.^12^

### Measurement of Functional Disability

The Katz index of independence in activities of daily living (Katz ADL)^13^ was used to assess the ability of participants to perform activities of daily living independently. The Katz ADL measures current functional ability, regardless of the cognitive functioning of the individual. It rates the participants' functional status by the adequacy of performance of six functions: bathing, dressing, toileting, transferring, continence, and feeding. The Katz ADL has reliability coefficients ranging from 0.87 to 0.94,^14^ and a concurrent validity of 0.95 when compared, for example, with the activity index.^15^ Each of the activities was rated as follows: (1) can do without difficulty; (2) can do with some difficulty; (3) can do only with assistance; (4) unable to do activity. We classified as disabled any respondent with an ADL score greater than or equal to 2. A subgroup of 37 respondents was assessed twice, about 7 days apart, to determine the test–retest reliability of these disability markers. Agreement was generally very good to excellent, with a κ range of 0.65 to 1.0. The instrument was translated into the local Yoruba language, using the iterative back-translation method, and subjected to cultural adaptation.

### Other Data

Participants were asked their age (in years) and the number of years of formal education attained in their lifetime. In cases where records of birth were not readily available, estimates of age were determined using a previously validated list of historical events.^16^

Residence was classified as rural (<12,000 households), semi-urban (12,000–20,000 households) or urban (>20,000 households) based on the Nigerian census categorization at the time of study. Economic status was estimated using an inventory of 21 household and personal items,^17^ and calculated as the ratio of the total number of possessions they had relative to the median number of possessions of the overall sample, and classified as low (≤0.5); low-average (>0.5–1.0); high-average (>1.0–2.0), or high (>2). For reasons of low sample sizes of the higher economic categories, high-average and high were merged to form a single category.

The presence of chronic diseases such as arthritis, stroke, angina, diabetes, chronic lung disease, or cancer was ascertained using standard symptom-based questions.^18^ Participants were also asked whether, in the past year, they were able to access healthcare when it was needed. Use of tobacco and alcohol was categorized, based on self-report, as ever having smoked or not, and ever used alcohol or not. Those who responded in the affirmative to ever using alcohol were further classified into regular (weekly use or more often) or occasional users (less often than weekly use).

Social network was assessed with the relevant items in the CIDI. The items enquire about the frequency of respondent's contact with family members who do not live with the respondent as well as the frequency of contact with friends. In this report, we have dichotomized the responses into less than once in 6 months and those that are more than once in 6 months. Social participation was assessed using items derived from the WHO Disability Assessment Schedule, version 2.^19^ Participants were asked the following two questions: “‘During the last 30 days, how much did you join in family activities such as eating together, talking with family members, visiting family members, working together?’” and “‘During the last 30 days, how much did you join in community activities such as festivities, religious activities, talking with community members, working together?’” Answers were rated as 1 (not at all), 2 (a little bit), 3 (quite a bit), and 4 (a lot). In this report, participants who answered “not at all'' to either question were rated as having poor social participation.

#### Ascertainment of Dementia

The adapted 10-Word Delay Recall Test learning list (10-WDRT) was used to screen for dementia at baseline and each follow-up wave. In participants scoring less than 2 on the 10-WDRT, a second-stage assessment was conducted with the use of the Clinician Home-based Interview to assess Function (CHIF).^20^ Subsequently, a psychiatrist reviewed all available information to determine the presence or absence of dementia. The information included scores on the 10-WDRT (<2) and CHIF (<18), interviewer's observations, reported functional status, as well as the temporal relationship of the onset of any co-occurring depressive disorder. A complete description of the dementia ascertainment procedure in the ISA is presented in the supplemental material (see text documents, supplemental digital object 1).

### Definition of MDD Trajectory Groups

**Figure 1:**
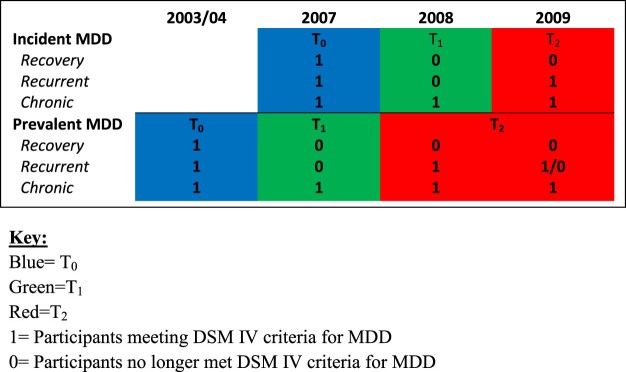
Timelines and courses of prevalent and incident major depressive disorder (MDD).

We grouped together prevalent (in 2003–2004) and incident (in 2007) MDD participants to increase our sample size (Figure 1). Participants who did not meet MDD criteria in any subsequent waves were considered to have recovered from MDD (symptomatic remission [SR]). Those still presenting with MDD criteria in subsequent waves were considered to have a chronic/recurrent course (CR).^21^ Incident cases (in 2007) were the subjects meeting criteria for MDD for the first time in the corresponding (2007) wave of follow-up. These were determined after censoring prevalent cases of MDD in 2003–2004. Cases with dementia, ascertained as previously described, were also excluded in ascertaining incident cases of MDD in 2007.

### Statistical Methods

We compared the sociodemographic characteristics of participants who could not be followed up in 2009 with those who were followed up using design-based F tests. Using the same tests, we also compared the baseline characteristics of participants across the SR and CR groups (Table 1). In conducting these comparisons, we implemented corrections accounting for survey design.^22^

**Table 1:** Baseline Characteristics of Participants in the Major Depressive Disorder-Trajectory Groups

Characteristic	Chronic/Recurrent N = 116 (%)	Recovered N = 85 (%)	Design-Based F statistic F_(d1,d2)_ = F	p
***Age, years***				
** 65–69**	43 (40.5)	26 (33.6)	F_(2.48, 71.89)_ = 0.54	0.625
** 70–74**	30 (31.1)	21 (40.0)		
** 75–79**	16 (16.9)	14 (17.2)		
** 80** **+**	27 (11.5)	24 (9.3)		
***Sex***				
** Male**	47 (49.3)	31 (52.9)	F_(1, 29)_ = 0.1953	0.662
** Female**	69 (50.7)	54 (47.1)		
***Residence***				
** Urban**	33 (26.5)	27 (28.1)	F_(1.94, 56.22)_ = 1.12	0.338
** Semi-urban**	49 (48.5)	27 (35.6)		
** Rural**	34 (25.0)	31 (36.3)		
***Education, years***				
** ** **≥** **7**	18 (19.3)	18 (20.7)	F_(1.82, 52.80)_ = 0.03	0.962
** 1–6**	29 (28.1)	23 (27.9)		
** 0**	69 (52.7)	44 (51.4)		
***Socioeconomic status***				
**High or high-average**	46 (51.1)	33 (48.3)	F_(1.85, 53.56)_ = 0.05	0.938
** Low-average**	43 (32.6)	28 (34.6)		
** Low**	27 (16.3)	24 (17.2)		
***Marital status***				
**Separated**^a^	58 (44.1)	46 (37.5)	F_(1, 29)_ = 0.6639	0.422
**Currently married**	58 (55.7)	39 (62.5)		
***Tobacco smoking***^b^				
** Ever**	42 (38.9)	27 (44.5)	F_(1, 29)_ = 0.4327	0.516
** Never**	74 (61.1)	58 (55.4)		
***Alcohol use***^b^				
** Regular (at least weekly)**	19 (19.3)	11 (19.0)	F_(1.87, 54.14)_ = 0.029	0.965
Occasional (<weekly)	33 (27. 1)	22 (29.8)		
** Never**	64 (53.6)	52 (51.2)		
***Comorbid medical conditions***				
** Yes**	52 (47.4)	26 (37.1)	F_(1, 29)_ = 1.144	0.294
** No**	64 (52.6)	59 (62.9)		
***Accessing of healthcare***^c^				
** Yes**	64 (59.3)	50 (60.6)	F_(1.73, 50.06)_ = 0.070	0.909
** No**	19 (15.4)	11 (16.6)		
** Unknown**	33 (25.4)	24 (22.8)		
***Social contacts***^b^				
>once in 6 months	101 (91.1)	70 (86.9)	F_(1, 29)_ = 0.727	0.401
<once in 6 months	15 (8.9)	15 (13.1)		
***Social participation***^b^				
** Good**	102 (89.8)	71 (88.5)	F_(1, 29)_ = 0.093	0.763
** Poor**	14 (10.2)	14 (11.5)		

a Separated by death or divorce.

b For simplicity, missing values were imputed using mode imputation method (for tobacco smoking N = 10, for alcohol use N = 10, for social contacts N = 8, for social participation N = 6).

c Whether participant accessed healthcare when it was needed for any health condition in the past year.

For the purpose of investigating the factors associated with SR in the study we conducted weighted multiple logistic regression analyses with SR as the dependent variable. Age, sex, and economic status were included as covariates in adjusted models. Odds ratios (ORs) with 95% confidence intervals (Cis) for this analysis are presented in Table 2.

**Table 2:** Association of Baseline Characteristics with Recovery from Major Depressive Disorder in the Ibadan Study of Ageing (N = 201)

Characteristic	Unadjusted OR (95% CI)	p^c^	Adjusted OR (95% CI)^d^	p^c^
***Age, years***				
** 70–74**	1.6 (0.8, 3.2)	0.600	1.6 (0.7, 3.2)	0.639
** 75–79**	1.2 (0.4, 3.7)		1.2 (0.4, 3.8)	
** 80** **+**	1.0 (0.4, 2.3)		1.0 (0.4, 2.3)	
***Sex***				
** Female**	0.9 (0.4, 1.7)	0.662	0.9 (0.4, 1.8)	0.662
***Residence***				
** Semi-urban**	0.7 (0.3, 1.7)	0.402	0.8 (0.2, 1.4)	0.281
** Rural**	1.3 (0.5, 3.8)		1.2 (0.4–3.7)	
***Education, years***				
** ** **≥** **7**	1.1 (0.4, 2.8)	0.978	1.2 (0.5, 3.0)	0.921
** 1–6**	1.0 (0.5, 2.0)		1.0 (0.5, 2.1)	
***Socioeconomic status***				
**Low**	1.0 (0.3, 3.1)	0.942	0.9 (0.3, 3.0)	0.894
**Low-average**	0.9 (0.4, 2.1)		0.8 (0.3, 2.2)	
***Marital status***				
** Currently married**	1.3 (0.7, 2.7)	0.422	1.4 (0.7, 3.2)	0.347
***Tobacco smoking***^a^				
** Ever**	1.3 (0.6, 2.6)	0.516	1.3 (0.6, 2.9)	0.580
***Alcohol use***^a^				
**Occasional (<weekly)**	1.1 (0.4, 3.4)	0.963	1.2 (0.3, 4.0)	0.965
** Never**	1.0 (0.3, 3.4)		1.1 (0.3, 4.0)	
***Comorbid medical conditions***				
** No**	1.5 (0.7, 3.5)	0.295	1.7 (0.7, 4.0)	0.239
***Accessing healthcare***^b^				
** Yes**	0.9 (0.4, 2.0)	0.934	1.1 (0.5, 2.4)	0.922
** Unknown**	0.8 (0.3, 2.6)		0.9 (0.3, 2.6)	
***Social contacts***^a^				
** ** **>** **once in 6 months**	0.7 (0.2, 1.9)	0.403	0.6 (0.2, 1.7)	0.297
***Social participation***^a^				
** Good**	0.9 (0.4, 2.1)	0.763	0.8 (0.3, 2.0)	0.622

*Notes:* Reference groups are age 65–69, male, urban site, 0 year of education, high or high-average economic status,never smoker, regular drinker, no access to healthcare, no social contact, and no social participation. CI: confidence interval; OR: odds ratio.

a For simplicity, missing values were imputed using mode imputation method (for tobacco smoking N = 10, for alcohol use n = 10, for social contacts N = 8, for social participation N = 6).

b Whether participant accessed healthcare when it was needed for any health condition in the past year.

c p values are based on adjusted Wald test using F statistics with design degrees of freedom (df) where the numerator df is the number of coefficients for that covariate (e.g., for age df = 3) and the denominator df equals 29.

d Adjusted models control for age, sex, and socioeconomic status.

We used mixed effects linear regression models with a random intercept in examining the association between the types of MDD trajectories and ADL functioning during the 5 years of follow-up. In these models,^23^ ADL functioning was the dependent variable. The mixed effects model accounts for correlations between observations within the same person and it can be used when all repeated data are not available for each participant. As we were more interested in functional recovery in the SR group, we compared ADL functioning between two groups (SR and CR). The groups, along with time, as well as group and time interaction, age, sex and socioeconomic status were entered as fixed factors.

Data were analyzed using Stata version 14.0.^24^ The *survey* commands in Stata were used to account for the study sampling scheme and the *mixed* command was used for the linear mixed effects models. A significance level of 0.05 was used throughout the analyses.

## Results

### Study Sample

**Figure 2:**
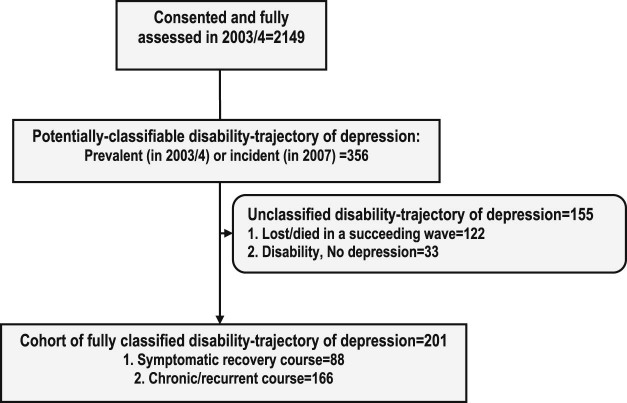
Flow chart showing the characterization depression and disability courses in the study.

In Figure 2, 2,149 participants were fully assessed in 2003–2004. Of this number, it was possible to classify 201 participants into two trajectories of MDD and disability as follows: SR (85 participants) and CR (116 participants).The baseline characteristics of participants belonging in the two groups are presented in Table 1. Their mean age was 74.2 (SD: 8.2) years. Complete follow-up data were available for 90% of participants in the trajectory groups. Those who died or were lost to follow-up were more likely to belong to the lowest age category (65–69 years). No other baseline characteristics significantly differentiated those who died or were lost to follow-up from those who completed the study.

### Association of SR with Baseline Characteristics and Course of Disability in MDD

In Table 2, we see that SR was not associated with baseline factors investigated in weighted multiple logistic regression analyses.

Table 3 shows that ADL functioning in SR participants worsened across the years of follow-up in mixed-effect linear regression (β = 1.0, 95% CI: 0.2, 1.8). Poorer ADL was predicted by age (β = 2.9, 95% CI: 1.8, 4.0) and economic status (β = 1.4, 95% CI: 0.3, 2.4).

**Table 3:** Mixed Effects Linear Models for ADL in the Depression Trajectory Groups

Characteristics	Coefficient	95% CI	p^a^	Degrees of Freedom
***Baseline: recovered vs. chronic/recurrent participants***	0.6	(−0.4, 1.6)	0.242	1
***Year effect among chronic/recurrent participants compared with their baseline***				
** 2007**	0.4	(−0.3, 1.1)	< .001	3
** 2008**	1.7	(1.0, 2.3)		
** 2009**	2.3	(1.6, 3.0)		
***Year effect among recovered participants compared with their baseline***				
** 2007**	1.0	(0.2, 1.8)	0.002	3
** 2008**	0.7	(−0.1, 1.5)		
** 2009**	1.0	(0.2, 1.8)		
***Age, years***				
** 70–74**	1.1	(0.7, 2.2)	<.001	3
** 75–79**	1.2	(−0.03, 2.5)		
** 80** **+**	2.9	(1.8, 4.0)		
***Sex***				
** Female**	0.3	(−0.5, 1.2)	0.457	1
***Socioeconomic status***				
** Low**	1.1	(0.1, 2.2)	0.030	2
** Low-average**	1.4	(0.3, 2.4)		

*Notes:* Reference groups are age 65–69, male, high or high-average socioeconomic status. CI: confidence interval.

a p values are based on a χ^2^ test using the Wald test.

## Conclusions

The key findings of the present study were that despite symptomatic recovery from MDD, physical functioning deteriorated over time. Poorer physical functioning after symptomatic recovery from MDD was predicted by age and economic status.

The pattern of changes in physical functioning in the present study suggests that, following symptomatic recovery from late-life MDD, physical disability may linger and, in this population, even worsen over time. Only very few, non-experimental, prospective follow-up studies of community elderly populations have examined the phenomenon of whether recovery of physical functioning follows a different course to symptomatic recovery from MDD. Studies examining this question in the literature have rarely used standardized ascertainment procedures for MDD at baseline and follow-ups or included sufficient numbers of observations to be able to examine variability in MDD and disability over time. We identified one study examining 3-year trajectory of disability and depression in a large community sample of Americans who were participants in the Cardiovascular Health Study,^3^ and one secondary analysis of data pooled from four community-based prospective follow-up studies of Swedish elders.^4^ The other studies identified were conducted among primary care^25^ or day-clinic^26^ attendees. Similar to the present study, these studies^3^, ^4^, ^25^, ^26^ report that, in the context of late-life depression, there is a continuous course of disability despite symptomatic recovery. Also, other long-term follow-up studies, including those conducted in the general population, have found older age and low socioeconomic status as important determinants of impaired physical functioning in the context of depression.^27^

Our inability to demonstrate statistical association between baseline clinical–demographic factors and recovery from MDD was unexpected. This is because it has long been reported in both prospective studies investigating the natural history of late-life depression^28^, ^29^ and in treatment trials, such as those reviewed by Mitchell and Subramaniam (2005),^30^ that persons at a younger age are more likely to reach symptomatic remission, whereas those who are older may have a course of depression characterized by chronicity or recurrence of symptoms. Less than one-quarter of the cohort in the recovery trajectory group in the present study met the criteria for symptomatic recovery from MDD. This was due to a relatively high number of participants who were lost to follow-up after contributing depression and disability data. As such, we could only fully classify into the two depression trajectory groups about half of those who were potentially classifiable. The small sample size of participants in the SR group may have affected our ability to demonstrate an association between the baseline factors and recovery from MDD.

### Strengths and Limitations

It is important to highlight the important differences between this study and the other large, non-experimental, prospective longitudinal investigation of disability trajectories of late-life depression. First, this study, which is the first report of its kind in community-dwelling sub-Saharan African elders, is based on a fairly large sample of older adults derived from a wide geographical area (eight neighboring states in southwest and north-central regions of Nigeria—an area inhabited by 25 million people or about 22% of the Nigerian population at the time of study). Second, we have implemented a longer period of follow-up (2–5 years) and in multiple waves of observation than in previous examination of the phenomenon of recovery trajectory from depression in late-life. We identified one study examining 3-year trajectory of disability and depression in a large community sample of Americans who were participants in the Cardiovascular Health Study.^3^ Third, we used a standardized ascertainment procedure for depression and diagnosed MDD according to DSM-IV criteria. We note the plausible bidirectional relationship between depression and disability, where pre-existing disability may be considered a vulnerability marker for late-life depression^4^ and vice versa,^21^ with onset of depression serving as the tipping point of deteriorating disability. Viewed from this perspective, the question may arise as to whether, in the present study, the persisting and deteriorating disability found in the context of symptomatic recovery, did not, in fact, predate MDD, and therefore could not be the result of the syndrome. Although our index assessment of MDD did not exclude prior disability, functional disability was not a significant risk factor for incident MDD in our previous analyses examining the epidemiology of depression in the ISA.^5^ We are thus reasonably confident that the disability trajectory of MDD identified in the present study is part of the so-called recovery trajectory of depression,^31^ where disability may lag behind symptoms. As we have not investigated the potential influence of subsyndromal depression on ADL over time, however, we could not rule out the possibility that the pattern and sizes of change in ADL observed in the present study were not due to the intervening effect of sub-threshold depression. Similar to other large-scaled prospective longitudinal studies of this kind, we recorded attritions, which were mostly due to non-availability of some participants during the follow-up waves (many of the participants belonging in this category had relocated from the study location and their current addresses were either not known or could not be traced). There is no vital registration of deaths in Nigeria. As such, we could not be certain of the precise number of participants who could not be followed up as a result of their death. We therefore considered all those who could not be followed up as attritions. The result of this limitation is a reduction in the number of incident cases of MDD in 2007 and, consequently, the small size of the final sample that was classifiable into the two MDD trajectory groups. Even though we found that those who died or were lost to follow-up were more likely to belong to the lowest age category (65–69 years), we ensured that the effect of age was accounted for across our analyses.

To summarize, physical functioning deteriorated over time despite symptomatic recovery from late-life MDD in this representative sample of elderly persons living in a sub-Saharan African community. This finding has implications for policy and guidelines for the management of both depression and disability in late life. As disability in the context of late-life depression runs a continuous and deteriorating course, programs targeting functional disability in the elderly need to be considered, not only in terms of physical disorders, but also mental health disorders like depression. Secondly, future guidelines for treatment of late-life MDD should consider rehabilitation for any resulting functional disability. This may include implementation of symptomatic treatments for longer than currently recommended. These recommendations could be the basis for the design and clinical trial of future tailored interventions for late-life MDD in sub-Saharan Africa.
